# Supplementary material for: Effective Field Immobilisation and Capture of Giraffe (Giraffa camelopardalis)
Source: Animals (Basel). 2022 May 18;12(10):1290. doi: 10.3390/ani12101290 (PMC9137789; doi:10.3390/ani12101290)
Supplement: Supplementary file 1 [file animals-12-01290-s001.zip › animals-1648874-supplementary.pdf]

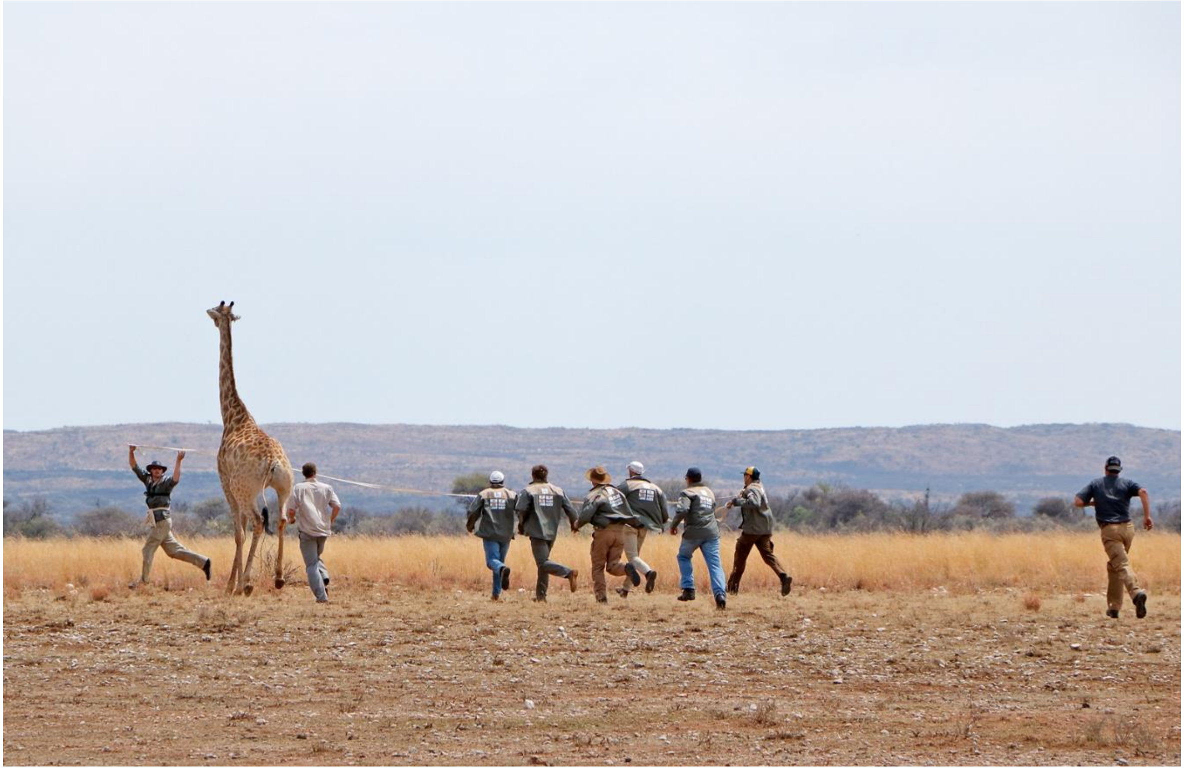

Figure S1: After the successful administration of the immobilisation drug/cocktail, the semi-immobilised giraffe should be caught and restrained by a supporting ground team.

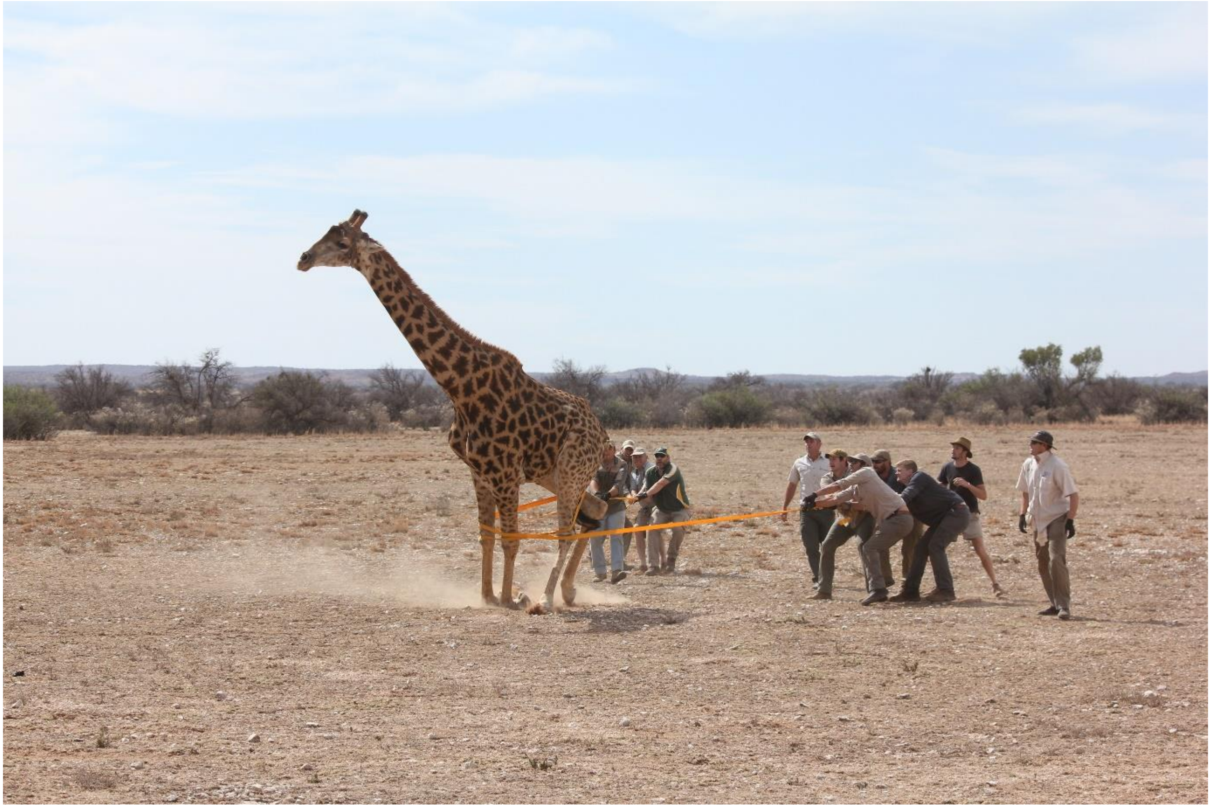

Figure S2: With the aid of a flat belt, held firmly in front of the animal (i.e., tripping), the ground team slows/restrains the animal until it falls to a prone position.

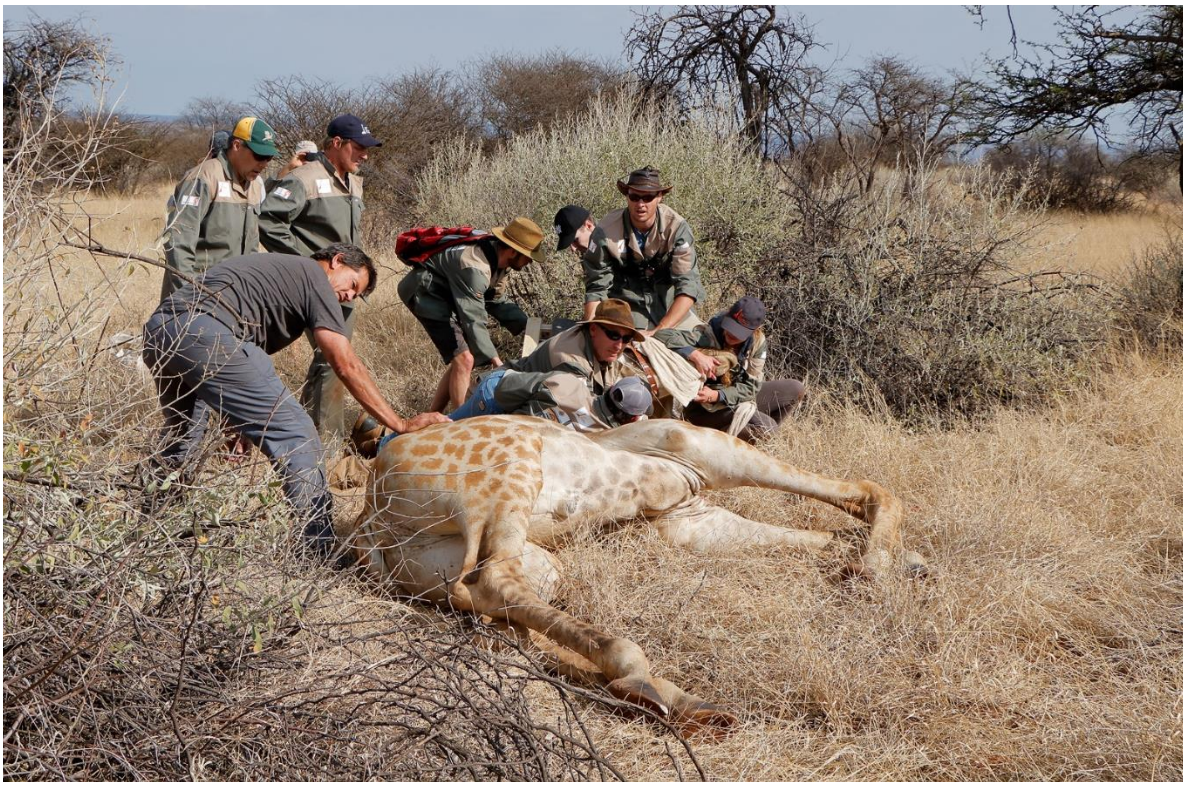

Figure S3: As soon as the immobilised giraffe is on the ground, the drug antagonist must be administered to prevent hypoxia.

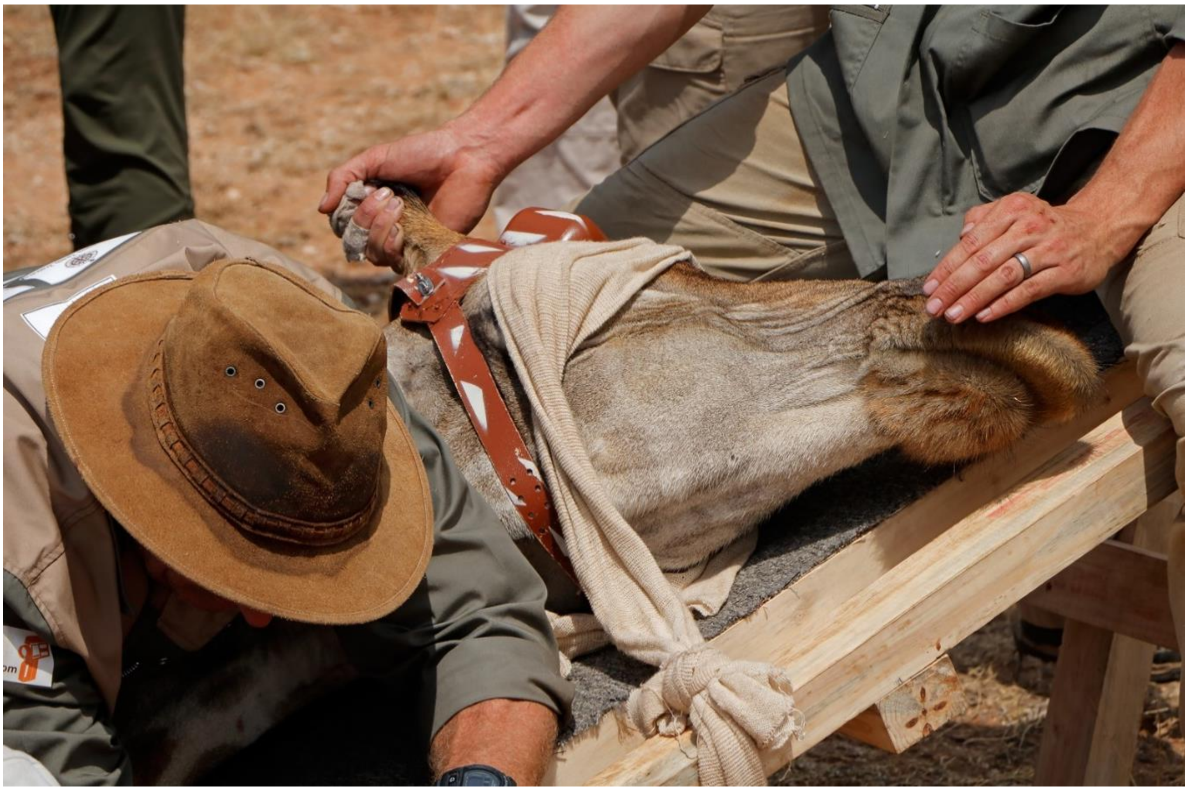

Figure S4: Breathing was monitored with the giraffe's head placed on the lap of a ground team member or mobile stretcher and having a firm grip on the ossicones. The eyes are covered and plugs are inserted into the ears to limit stimulation from external sources.

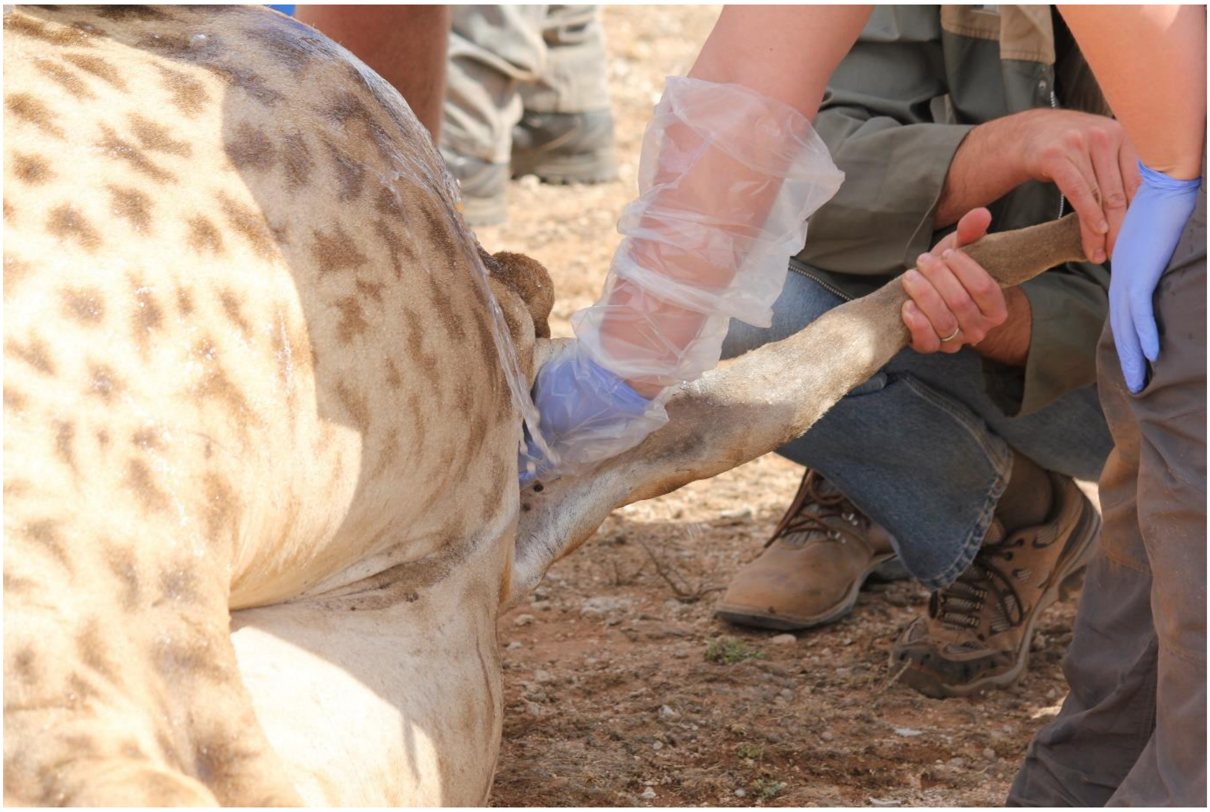

Figure S5: The normal body temperature was measured, by placing a digital thermometer deep within the rectum.

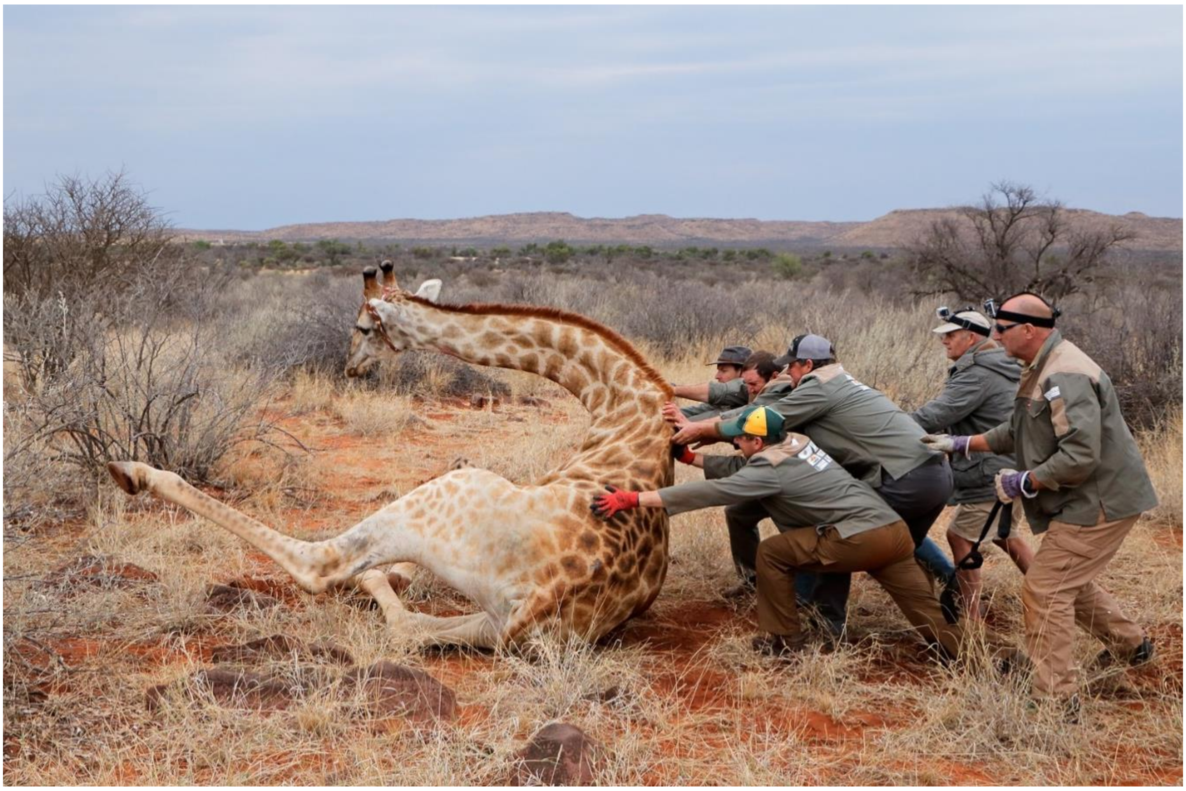

Figure S6: After data collection is finished the giraffe, which is now fully conscious, must be allowed to stand.

Recommendations for a standard operating procedure for giraffe immobilisation

With accumulated experience from over 75 consecutive successful immobilisation events, over a 10-year period, the following recommendations should be considered when attempting to immobilize giraffes.

- The successful capture of giraffes is hugely dependent on proper planning beforehand.
- The area and surrounding structures should be accounted for to ensure the safety of the animal once the administered drug takes effect. Where possible capture should always be attempted away from water on as flat as possible ground.
- A well-experienced capture team consisting of enough members contributes in making the capture a lot easier and significantly increases the safety of the animal and personnel.
- It is recommended that the capture team should consist of at least two veterinarians.
- Darting should not be performed from a distance of more than 40 m.
- The dart should be placed in the giraffe's shoulder rather than the rump as it provides a quicker knockdown time.
- When the immobilization of giraffes is attempted, the fastest possible knockdown time is crucial, as it reduces risk of possible self-inflicted harm.
- The preferred drug and dosages during this study was 14- and 18- mg of thiafentanil (A3080/Thianil) administered for females and males, respectively.
- During the present study, the ground veterinarian immediately injected the antagonist (100 mg Naltrexone directly into the jugular vein and 50 mg Naltrexone into the triceps muscle) to prevent hypoxia (respiratory depression).
- A 20-m polyester flat belt held firmly in front of the animal, was efficient in tripping the sedated animal to fall into a prone position.
- By making use of a mobile stretcher the head and upper neck of the immobilised giraffe was elevated to prevent passive regurgitation.
- During restraint, the monitoring of vital signs, such as the breathing rate and depth, body temperature, colour of the mucus membranes and heart rate, should be conducted.
- On a hot day, shade should be provided and water applied to the body of the giraffe to assist with the cooling of the animal.
- The animal's eyes should be blindfolded, and the ears should be plugged to minimize visual and audio stimulation. The blindfold will also assist in preventing the drying and hence damage to the eyes.
- The agent which was used for immobilisation should be fully reversed, before attempting to guide and load the animal, through the use of ropes, into a transportation crate.
- Giraffes transported in groups are much more relaxed than those transported in single crates.
- A short-acting tranquilizer should be administered during transport, to relieve the giraffe of stress and reduce any aggression towards other individuals in the same crate.
